# The dynamics of telomere length in primary and metastatic colorectal cancer lesions

**DOI:** 10.1038/s41598-023-35835-9

**Published:** 2023-06-05

**Authors:** Michal Kroupa, Ondrej Kubecek, Kristyna Tomasova, Petr Hanak, Marketa Krupova, Klara Cervena, Anna Siskova, Jachym Rosendorf, Petr Hosek, Ludmila Vodickova, Pavel Vodicka, Vaclav Liska, Stanislav John, Veronika Vymetalkova, Jiri Petera

**Affiliations:** 1 Department of Molecular Biology of Cancer, Institute of Experimental Medicine of the Czech Academy of Sciences, Videnska 1083, 142 20 Prague, Czech Republic; 2 Biomedical Centre, Faculty of Medicine in Pilsen Charles University, Alej Svobody 76, 323 00 Pilsen, Czech Republic; 3 Department of Oncology and Radiotherapy, Charles University, Medical Faculty and University Hospital in Hradec Kralove, Simkova 870, 500 38 Hradec Kralove, Czech Republic; 4 The Fingerland Department of Pathology, University Hospital in Hradec Kralove, Sokolska 581, 50005 Hradec Kralove, Czech Republic; 5 Institute of Biology and Medical Genetics, 1St Faculty of Medicine, Charles University, Albertov 4, 128 00 Prague, Czech Republic

**Keywords:** Tumour biomarkers, Colorectal cancer

## Abstract

Telomeric sequences, the structures comprised of hexanucleotide repeats and associated proteins, play a pivotal role in chromosome end protection and preservation of genomic stability. Herein we address telomere length (TL) dynamics in primary colorectal cancer (CRC) tumour tissues and corresponding liver metastases. TL was measured by multiplex monochrome real-time qPCR in paired samples of primary tumours and liver metastases along with non-cancerous reference tissues obtained from 51 patients diagnosed with metastatic CRC. Telomere shortening was observed in the majority of primary tumour tissues compared to non-cancerous mucosa (84.1%, p < 0.0001). Tumours located within the proximal colon had shorter TL than those in the rectum (p < 0.05). TL in liver metastases was not significantly different from that in primary tumours (p = 0.41). TL in metastatic tissue was shorter in the patients diagnosed with metachronous liver metastases than in those diagnosed with synchronous liver metastases (p = 0.03). The metastatic liver lesions size correlated with the TL in metastases (p < 0.05). Following the neoadjuvant treatment, the patients with rectal cancer had shortened telomeres in tumour tissue than prior to the therapy (p = 0.01). Patients with a TL ratio between tumour tissue and the adjacent non-cancerous mucosa of ≥ 0.387 were associated with increased overall survival (p = 0.01). This study provides insights into TL dynamics during progression of the disease. The results show TL differences in metastatic lesions and may help in clinical practice to predict the patient’s prognosis.

## Introduction

Colorectal cancer (CRC), the third most commonly diagnosed cancer in men and the second in women, represents a major public health challenge^1^. Detached CRC cells most frequently colonize the liver, as it is the first capillary bed encountered by the metastasizing cells via the portal vein^2^. Either at the time of diagnosis (synchronous disease) or during the follow-up period (metachronous disease), approximately 25% of patients are eventually diagnosed with disease recurrence in the liver^3^. Despite maximal surgical excision of the primary tumour, as well as metastases, the use of radiation, and chemotherapy, the 5-year relative survival rate for advanced-stage CRCs remains low (14% and 17% for colon and rectum, respectively)^4,5^. Understanding the molecular mechanisms influencing tumour growth and invasiveness, probability of recurrence, and treatment efficacy may lead to the development of novel and effective therapies to ameliorate the poor prognosis of late-stage cancers.

Nowadays, telomere maintenance mechanisms are established as targets in cancer treatment^6^. Telomeres, the specialized nucleoprotein structures located at the ends of chromosomes, are essential for genome stability^7,8^. Due to the “end-replication problem” phenomenon, telomeres shorten with each replication cycle^9^. When telomeres reach a critical length, they are considered dysfunctional, and the cell triggers apoptosis or the senescence state. Chromosomes lacking the telomere “capping structure” may get truncated and fused with other chromosomes possibly leading to the induction of chromosomal instability^10^ which represents a hallmark of cancer^11^. In addition, dysfunctional telomeres intrude on key cell-signalling pathways affecting cancer initiation and progression^12^.

To date, many studies have demonstrated the importance of telomeres as an outcome indicator and their association with distinct clinicopathological features of patients diagnosed with different cancers, including CRC^13–16^. However, the studies focusing on telomere dynamics from the primary tumour to metastasis in patients diagnosed with CRC are scarce^13,17,18^. We reported in our previous study telomere shortening in CRC liver metastases compared to primary tumors^13^. However, the number of patients in the study was a limiting factor^13^. Here, we hypothesized that telomere length (TL) assessed in either the primary tumours or metastases may help stratify the patients diagnosed with CRC into distinct molecular subgroups characterized by differences in survival and other clinicopathological characteristics.

In addition, since TL has the potential to be a predictive biomarker of clinical outcome to anti-epidermal growth factor receptor (EGFR) monoclonal antibody therapy in patients with Kirsten-ras (KRAS) wild-type metastatic CRC, we aimed to investigate whether KRAS oncogene mutations have an impact on telomere deregulation.

## Material and methods

### Formalin-fixed, paraffin-embedded samples (FFPE)

Fifty-one patients with histologically confirmed CRC and an available histological sample of CRC liver metastases were enrolled in this retrospective study. The median [interquartile range; IQR] age of the patients at the time of diagnosis of the primary tumour was 67.8 [62.2–72.0] years. All the patients developed either synchronous (n = 20) or metachronous (n = 31) CRC liver metastases. Moertel’s definition was used to distinguish between synchronous and metachronous metastases, i.e. those diagnosed within three months or more than three months from the primary tumour, respectively^19^. The patients were sampled for primary tumour (n = 51), the microscopically dissected adjacent non-cancerous mucosa (i.e. with no histological signs of dysplastic changes, carcinoma in situ or invasive cancer) located 1–2 cm either proximally (orally; n = 51) or distally (aborally; n = 19) from the primary tumour margin, liver metastasis (n = 51), and adjacent non-cancerous liver parenchyma (n = 51). Additionally, in six patients diagnosed with rectal cancer, both the tumour tissue sample prior and after the neoadjuvant therapy were used.

In total, eight patients within the studied group were treated with neoadjuvant therapy consisting of radiotherapy along with chemotherapy (bevacizumab or panitumumab in combination with fluoropyrimidines). Those patients undergoing neoadjuvant therapy were diagnosed with rectal cancer (n = 6; radiotherapy with [n = 5] or without [n = 1] concomitant fluoropyrimidine-based chemotherapy) and two with tumour located in the proximal colon.

Patient tissue samples were stored as paraffin-embedded blocks (FFPE) at the Fingerland Department of Pathology, University Hospital in Hradec Kralove. The samples were used for DNA isolation using Recover All Total Nucleic Acid Isolation Kit (Invitrogen, USA). The study was approved by the Ethics Committee of the Hradec Kralove University Hospital, Czech Republic (201207- S01P) and its implementation took place according to the approved conditions. Due to the retrospective nature of the study and because part of the patients had already died before the study initiation, the informed consent was waived by the Ethics Committee of the Hradec Kralove University Hospital, Czech Republic (201207- S01P). All experiments were performed in accordance with relevant guidelines and regulations.

### Telomere length measurement

TL was determined by a monochrome multiplex quantitative real-time PCR (qPCR) method, as described in detail previously and expressed as relative^13,20^. Briefly, Ct values for telomere sequences (T) and reference single copy gene (S; albumin) were determined simultaneously as a multiplex using QuantStudio™ 6 Flex Real-Time PCR System (Applied Biosystems, USA). All the samples were measured in triplicates. If a Ct deviation within a triplicate exceeded the value 0.3, the sample was remeasured. The standard curve was used to quantify telomere and albumin products based on the respective Ct values. The qPCR efficiency for telomere sequences varied between 100 to 107%, while the qPCR efficiency for the albumin gene ranged from 90 to 97%. The Ct data was normalized based on the qPCR efficiency as described previously^21^, and TL was expressed as the T/S ratio.

### *KRAS* mutation status

*KRAS* mutation status was assessed using either qPCR or Next Generation Sequencing (NGS). First, the DNA was extracted from paraffin-embedded tissue blocks by the Cobas DNA Sample Preparation Kit (Roche, Switzerland) according to the manufacturer´s protocol. In the case of qPCR, *KRAS* mutational status in exon 2 (codons 12 and 13), exon 3 (codons 59 and 61), and exon 4 (codons 117 and 146) was tested with the AmoyDx KRAS Mutation Detection Kit (Amoy Diagnostics, China). The analytical sensitivity given by manufacturers is the detection of 1% mutant alleles on a wild-type DNA background. In the case of NGS, the indexed Illumina NGS library was constructed from 100 ng tumour DNA by KAPA Hyper Plus Kit (Roche, Switzerland). The hybrid selection was performed with a custom KAPA Hyper Choice MAX Library (Roche, Switzerland). The library was designed using genome build hg19 NCBI Build 37.1/GRCh37, and the input genomic regions included *KRAS* (exons 2–5). Paired-end cluster generation and sequencing were performed according to standard protocols from Illumina, using MiniSeq kits. Sequencing data analysis and variant classification were performed by NextGENe software (Softgenetics, USA) with a minimum of 5% variant allele frequency filtering.

### Statistical analysis

The relationship between the age at diagnosis and TL was analysed by Kendall's tau non-parametric correlation. A possible association between TL and the sex of the patients was calculated by the Mann–Whitney *U* test. For the assessment of possible TL association with type 2 *Diabetes mellitus*, the distribution allowed the use of the Student’s *t* test.

TL differences between distinct tissues of each patient were analysed by the Wilcoxon Matched Pair test. Friedman's ANOVA (a non-parametric alternative to one-way repeated-measures ANOVA respecting the dependency of observations) and the Kruskal–Wallis test (ignoring the dependency of observations) were used to analyse telomere dynamics within all the samples including unpaired tissues. The TL differences between synchronous and metachronous metastases were assessed by the Mann–Whitney *U* test.

To analyse the relationship between TL and tumour localization, the patients were stratified into subgroups based on the primary tumour origin; proximal colon (diagnosis C18.0-C18.4, n = 14; including malignant neoplasm of the appendix, caecum, ascending colon, hepatic flexure, and transverses colon), distal colon (diagnosis C18.5-C19, n = 22; including malignant neoplasm of the splenic flexure, descending colon, sigmoid colon and rectosigmoid junction) and rectum (diagnosis C20, n = 15). Differences between individual subgroups of patients were determined by the Kruskal–Wallis ANOVA.

For survival analysis, overall survival (OS) was defined as the time from the surgery of the primary tumour to the date of death or the date of the last follow-up, and metastatic CRC OS was defined as the time from the surgery of the liver metastasis to the date of death or the date of the last follow-up. The Cox proportional hazards model was used to explore the associations of TL with these survival variables. All of the possible TL-related survival predictors were tested both as measured and after Box-Cox transformation to compensate for possibly non-normal distributions. In order to visualise these associations with Kaplan–Meier plots, a threshold value was determined for each prognostic variable and the patients were stratified into two groups accordingly. Each threshold was identified through an automated optimization process implemented in Matlab (2019a, MathWorks Inc., USA), in which the threshold value producing the smallest Cox-Mantel p-value was determined and selected.

All reported p-values are two-tailed, and the level of statistical significance was set at α = 0.05. Statistical analysis was performed in Statistica (ver. 12 Cz, TIBCO Software Inc., USA).

## Results

All the results along with the clinicopathological details of the patients are summarized in Table 1.

**Table 1:** TL values and clinicopathological characteristics of the studied population.

	Tumour tissue, median TL [IQR]; N	Adjacent non-cancerous mucosa located orally, median TL [IQR]; N	Adjacent non-cancerous mucosa located aborally, median TL [IQR]; N	Liver metastatic tissue, median TL [IQR]; N	Non-cancerous liver tissue, median TL [IQR]; N
Studied group of patients	N = 51	N = 51	N = 19	N = 51	N = 51
Samples with successfully measured TL	2.01 [1.57–2.67]; N = 48	2.96 [2.17–4.46]; N = 44	3.10 [2.59–4.50]; N = 19	1.85 [1–47-2.95]; N = 49	2.46 [2.03–3.36]; N = 45
Tissue pairs comparison	1.91 [1.54–2.51]; N = 44	2.96 [2.17–4.46]; N = 44			
	Ref	P < 0.0001*			
	1.84 [1.58–2.79]; N = 19		3.10 [2.59–4.50]; N = 19		
	Ref		P = 0.003*		
	2.05 [1.56–2.72]; N = 45			1.76 [1.47–2.95]; N = 45	
	Ref			P = 0.407	
				1.81 [1.45–2.97]; N = 42	2.49 [2.02–3.37]; N = 42
				Ref	P = 0.023*
		3.18 [2.17–4.61]; N = 42		1.73 [1.45–2.81]; N = 42	
		Ref		P = 0.0001*	
Sex					
Male	2.13 [1.65–2.88]; N = 27	2.96 [2.12–5.05]; N = 24	3.95 [3.06–8.46]; N = 10	1.76 [1.49–2.10]; N = 28	2.30 [2.06–3.01]; N = 24
Female	1.84 [1.54–2.52]; N = 21	3.11 [2.28–4.05]; N = 20	2.81 [2.59–3.40]; N = 9	2.20 [1.41–3.03]; N = 21	2.57 [1.98–3.37]; N = 21
	P = 0.547	P = 0.878	P = 0.111	P = 0.664	P = 0.641
Tumor localisation					
Colon; C18	1.71 [1.10–2.20]; N = 22	2.26 [1.77–4.22]; N = 19	3.10 [2.67–4.50]; N = 9	1.86 [1.57–2.12]; N = 24	2.51 [2.24–3-17]; N = 21
Rectosigmoid junction; C19	2.37 [1.57–4.12]; N = 11	3.93 [2.15–5.48]; N = 10	2.81 [2.37–8.52]; N = 3	1.69 [1.41–3.03]; N = 11	2.81 [2.31–3.36]; N = 9
Rectum; C20	2.20 [1.76–3.32]; N = 15	2.93 [2.71–4.62]; N = 15	3.40 [2.59–4.23]; N = 7	1.91 [1.47–3.63]; N = 14	2.10 [1.36–3.61]; N = 15
	P = 0.031	P = 0.214	P = 0.944		
Proximal colon; C18.0-C18.4	1.67 [1.05–1.83]; N = 12	2.20 [1.73–3.45]; N = 12	2.67 [2.52–8.46]; N = 5	1.76 [1.61–2.01]; N = 13	2.40 [1.93–2.95]; N = 12
Distal colon; C18.5-C19	2.19 [1.57–2.78]; N = 21	3.80 [2.15–4.61]; N = 17	3.10 [2.81–4.50]; N = 7	1.90 [1.41–2.95]; N = 22	2.82 [2.32–3.68]; N = 18
Rectum; C20	2.20 [1.76–3.32]; N = 15	2.93 [2.71–4.62]; N = 15	3.40 [2.59–4.23]; N = 7	1.91 [1.47–3.63]; N = 14	2.10 [1.36–3.61]; N = 15
	P = 0.043*	P = 0.155	P = 0.961		
C18.0-C18.4 *vs*. C20	P < 0.05*				
Neoadjuvant therapy					
YES	2.16 [1.86–2.43]; N = 8	2.51 [2.04–3.23]; N = 8	3.06 [3.06–3.06]; N = 1	2.07 [1.52–2.94]; N = 8	2.00 [1.36–2.22]; N = 6
NO	1.85 [1.55–2.83]; N = 40	3.45 [2.17–4.61]; N = 36	3.25 [2.59–4.50]; N = 18	1.76 [1.47–2.95]; N = 41	2.57 [2.10–3.37]; N = 39
	P = 0.875	P = 0.313			
YES; TL ratio—Tumor/ Adjacent mucosa (located orally)	0.71 [0.59–1.12]; N = 8				
NO; TL ratio—Tumor/ Adjacent mucosa (located orally)	0.60 [0.46–0.80]; N = 40				
	P = 0.022*				
Rectum; C20 prior to neoadjuvant therapy	5.10 [3.44–17.42]; N = 6				
Rectum; C20 following neoadjuvant therapy	2.01 [1.56–2.27]; N = 6				
	P = 0.012*				
Relapse—metastatic disease					
Metachronous	1.96 [1.46–2.78]; N = 30	3.42 [2.14–4.61]; N = 28	3.40 [2.81–4.92]; N = 13	1.59 [1.33–2.76]; N = 30	2.76 [2.03–3.64]; N = 27
Synchronous	2.04 [1.69–2,56]; N = 18	2.83 [2.20–3.96]; N = 16	2.85 [2.52–4.23]; N = 6	2.01 [1.75–3.59]; N = 19	2.31 [1.98–3.09]; N = 18
	P = 0.616			P = 0.034*	
*KRAS*					
Wild-type	2.20 [1.58–2.98]; N = 19	3.71 [2.86–4.62]; N = 17	3.10 [2.95–4.50]; N = 9	2.00 [1.56–3.63]; N = 22	2.51 [2.10–3.37]; N = 21
Mutation	1.87 [1.31–2.56]; N = 13	3.09 [1.89–5.19]; N = 12	3.30 [2.39–10.99]; N = 4	1.86 [1.41–2.11]; N = 12	2.76 [2.28–3.82]; N = 11
	P = 0.173				
Type 2 *Diabetes mellitus*					
YES	2.02 [1.69–2.37]; N = 10	2.86 [1.82–3.60]; N = 10	4.23 [2.67–4.50]; N = 3	1.54 [1.26–2.12]; N = 12	2.20 [1.53–2.57]; N = 10
NO	2.01 [1.54–2.78]; N = 38	3.23 [2.18–4.62]; N = 34	3.08 [2.55–4.36]; N = 16	1.96 [1.56–3.03]; N = 37	2.76 [2.10–3.64]; N = 35
	P = 0.75	P = 0.73			P = 0.037*

Significant values are in bold.

Relative TL was successfully measured in 48 samples of the primary tumour, 63 samples of adjacent non-cancerous mucosa (sampled either orally (n = 44) or aborally (n = 19) from the primary tumour), 49 samples of liver metastasis, and 45 samples of non-cancerous liver parenchyma.

The comparisons in the Results section are based on respective tissue pairs comprising primary tumors and adjacent colorectal mucosa; and colorectal liver metastases and either colorectal mucosa or non-cancerous liver parenchyma. The primary tumor-adjacent mucosa pairs were on disposal both from patients with and without neoadjuvant treatment. TL in any investigated tissue was not correlated with patients´ age or sex. Therefore, the results are based on age-non-adjusted TL data.

### Comparisons of telomere length in distinct tissues of the patients

TL measured in primary tumour tissues (n = 44, 1.91 [1.54–2.51]) was significantly shorter in comparison with TL assessed in the orally sampled adjacent non-cancerous mucosa (n = 44, 2.96 [2.17–4.46], Wilcoxon Matched Pairs Test, p < 0.0001). TL was also shorter in tumour tissue (n = 19, 1.84 [1.58–2.79]) than in the aborally sampled non-cancerous mucosa (n = 19, 3.10 [2.59–4.50], Wilcoxon Matched Pairs Test, p = 0.003). This phenomenon was documented in a high percentage of tumours compared with orally or aborally sampled non-cancerous mucosa counterparts (84.1% and 89.5% respectively).

No statistically significant TL differences were observed between paired primary tumours (n = 45, 2.05 [1.56–2.72]) and liver metastases (n = 45, 1.76 [1.47–2.95], Wilcoxon Matched Pairs Test; p = 0.407). Shorter telomeres were documented in 53.3% of metastases compared to primary tumours. However, TL measured in liver metastases was statistically significantly shorter compared to the non-cancerous liver parenchyma (n = 42, 2.49 [2.02–3.37]; Wilcoxon Matched Pairs Test, p = 0.023). Such TL shortening was documented in 66.7% of metastases. Furthermore, TL was shorter in liver metastases (n = 42, 1.73 [1.45–2.81]) compared to non-cancerous colon mucosa tissue samples (n = 42, 3.18 [2.17–4.61], Wilcoxon Matched Pairs Test, p = 0.0001).

**Figure 1:**
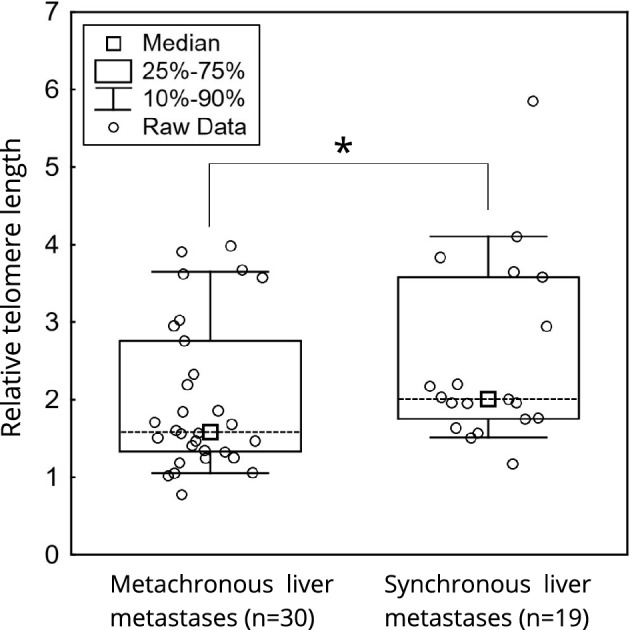
Comparison of TL between metachronous and synchronous liver metastases (p = 0.034).

The patients diagnosed with metachronous liver metastases displayed shortened TL in the metastatic tissue (n = 30, 1.59 [1.33–2.76]) compared to the patients diagnosed with synchronous liver metastases (n = 19, 2.01 [1.75–3.59], Mann–Whitney U test; p = 0.034, Fig. 1). However, the time interval between the diagnosis of the primary tumour and relapse correlated with neither TL in tumour tissue (n = 47, Kendall Tau Correlation, p = 0.56) nor tumour to non-cancerous mucosa TL ratio (n = 45, Kendall Tau Correlation, p = 0.60).

**Figure 2:**
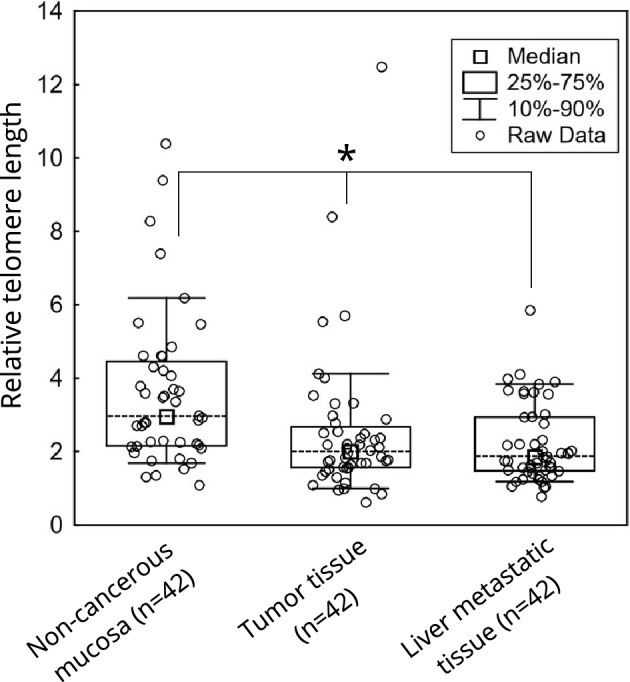
Analysis of telomere dynamics from non-cancerous mucosa to liver metastases over primary tumour tissue (p < 0.0001).

TL dynamics (the change in TL from non-cancerous mucosa to primary tumour and metastasis) was analysed by Friedman ANOVA (n = 42, TL values within paired tissues only where TL data were available for all three tissues, Fig. 2) and Kruskal–Wallis test (n = 141 non-paired tissues (including all the tumours, non-cancerous mucosa and metastatic liver tissues)). Both comparisons showed difference in TL within the groups; p < 0.0001 and p = 0.0010, respectively.

### Telomere length in tumors differs by their tumour localization

**Figure 3:**
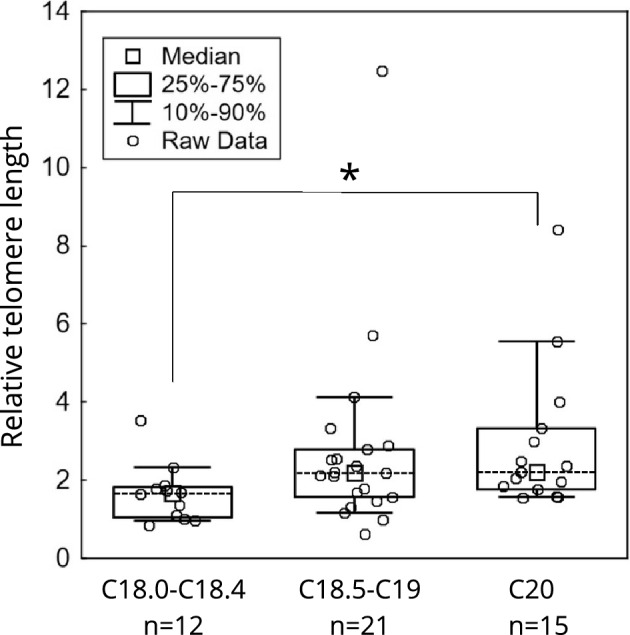
Comparison of TL in primary tumour tissue based on tumour localization. Tumours in proximal site of the colon *vs*. rectal cancer (p < 0.05).

The patients diagnosed with tumours located within the proximal colon (diagnosis C18.0-C18.4) had shorter TL in tumour tissue (n = 12, 1.67 [1.05–1.83]) than patients diagnosed with malignant neoplasm of the rectum (C20, n = 15, 2.20 [1.76–3.32]; Kruskal–Wallis test, p < 0.05, Fig. 3). No TL differences between the distal colon tumours (diagnosis C18.5-C19, n = 21), and the proximal colon or rectal tumours were found (p values not shown).

### Impact of neoadjuvant therapy on telomere length

Irrespective of tumour localization, the patients undergoing the neoadjuvant therapy had higher tumour to non-cancerous mucosa TL ratio (n = 8, 0.71 [0.59–1.12]) than the individuals without neoadjuvant therapy (n = 40, 0.60 [0.46–0.80], p = 0.022). In addition, tumour tissues obtained from the patients diagnosed with malignant neoplasm of the rectum had significantly shorter TL following the neoadjuvant therapy (n = 6, 2.01 [1.56–2.27]) than the tumours of the same individuals sampled prior to the neoadjuvant therapy treatment (n = 6, 5.10 [3.44–17.42]; Wilcoxon Matched Pairs Test, p = 0.012). Telomere shortening after the therapy was, in these samples, documented in 100% of the tumours.

### Influence of telomere length on patient survival

The median overall survival (OS) in the group of patients was 5.88 years from the diagnosis of the primary tumour and 3.82 years from the diagnosis of liver metastasis. The three-year and 5-year overall survival rates were 86.87% (95% CI 77.70–96.04) and 61.93% (95% CI 47.36–76.50), respectively. The 3- and 5-year survival rates of all patients from the diagnosis of relapse were 61.36% (95% CI 47.76–74.96) and 43.06% (95% CI 22.84–63.27), respectively.

**Figure 4:**
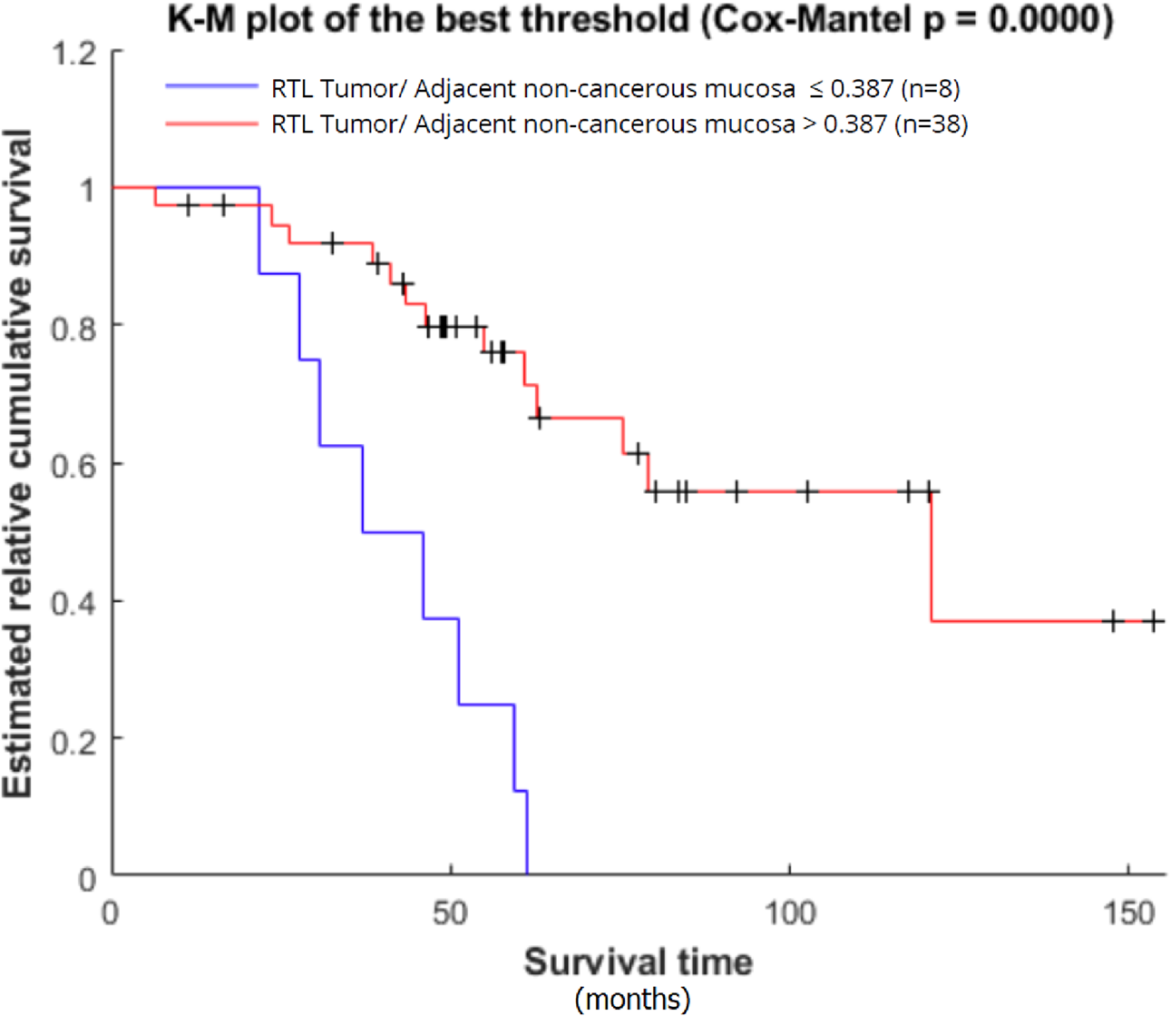
Comparison of OS between the patients with tumour to adjacent mucosa TL ratio > 0.387 and ≤ 0.387 (p < 0.0001).

There was no difference in OS while stratifying all the patients based on median TL measured in all investigated tissues. However, a higher tumour to adjacent mucosa TL ratio (n = 38; ≥ 0.387) was significantly associated with prolonged OS (n = 46, p = 0.01, Hazard rate = HR = 0.12, 95% CI HR 0.01–0.95). After automated stratification, patients with tumor TL lower or equal to 38.6% of the corresponding non-cancerous mucosa TL (TL ratio ≤ 0.386; lower quartile Q1; n = 8) clearly showed worse OS compared to those with higher TL ratio values (n = 38, p < 0.0001, Fig. 4).

A similar result was not observed between liver metastasis to liver parenchyma TL ratio and metastatic CRC OS (time from the diagnosis of remission to death; p = 0.91).

### Other clinical parameters investigated in connection with telomere length

#### Telomere length in *KRAS*-mutant tumors

The patients with *KRAS* mutations (n = 13, 1.87 [1.31–2.56]) did not show statistically significant different TL in tumour tissue compared to the individuals with *KRAS* wild-type status (n = 19, 2.20 [1.58–2.98], T-test, p = 0.173).

#### The size of metastatic lesions corresponds with telomere length

The liver metastatic lesion size correlated with the TL measured in metastases (n = 47, Kendall Tau Correlation, **p = 0.048**).

#### The effect of BMI and type 2 diabetes mellitus on telomere length

There was no relationship between either sex or BMI of the individuals and TL measured within any investigated tissue (p values not shown). The patients diagnosed with type 2 *Diabetes mellitus* had shorter TL values in liver parenchyma (n = 10, 2.20 [1.53–2.57]) than the individuals without the same diagnosis (n = 35, 2.76 [2.10–3.64]; T-test p = 0.037).

## Discussion

In this study, we determined TL in primary CRC tissues and corresponding liver metastases along with the non-cancerous colon mucosa and liver parenchyma tissue samples to study the TL dynamics during the disease progression.

We confirmed that primary CRC tissue exhibits shorter TL compared to non-cancerous reference mucosa, which has been previously described by us and many other studies^13,17,22–26^. This phenomenon strongly supports the notion that the telomere crisis might be one of the pivotal events in the disease development^24^. Interestingly, a minor group of patients (n = 7) in our study displayed no apparent change in TL or evinced even slight telomere elongation in tumour tissue compared to non-cancerous mucosa. All patients within this group were diagnosed with either rectal cancer or cancer of the rectosigmoid junction. Based on this observation we decided to elucidate whether TL in primary tumour tissue is related to tumour localization. Following the analysis, longer telomeres were observed in tumours of rectal cancer patients than in patients diagnosed with tumours located in the proximal colon. This observation is in agreement with our previous study, including a group of 721 CRC patients^13^. Uneven TL between tumours in the colon and rectum may be, in theory, impacted by different embryological origins and functions of both bowel segments^27,28^. However, other studies did not find similar variance comparing TL within distinct segments of the bowel^17,26^.

Interestingly, we did not find any TL differences between primary tumours and liver metastases (n = 45). To date, hardly any studies have focused on the TL evaluation in both primary tumour tissues and metastatic lesions in patients diagnosed with CRC. Suraweera et al*.* documented telomere lengthening in metastatic tissues contrary to primary tumours of 11 patients diagnosed with CRC^18^. Unfortunately, the authors did not provide details of the metastatic patients. On the other hand, we found telomere shortening in primary tumours compared to metastatic tissues in our previous study, where the availability of both tissues was from 12 CRC patients diagnosed solely with either left-sided or rectal tumors^13^. We were not able to make any comparisons of the present results with the aforementioned studies, especially due to the missing or uneven characteristics of the metastatic CRC patients. Therefore, further large-scale studies are needed to elucidate the TL status during the progression of CRC disease.

In addition, we have documented shorter telomeres in metachronous metastatic tissues compared to synchronous liver metastases. Previous studies described the stimulation of epithelial to mesenchymal transition (EMT) by telomerase activity and induction of cancer cell stemness in various tumours, including CRC^29,30^. We can only speculate whether CRC cell subpopulations with higher telomerase activity could be more prone to develop early metastases via EMT and be responsible for synchronous liver metastases. Unfortunately, we were unable to confirm this hypothesis in our studied group of patients due to the suboptimal quality of the RNA derived from FFPE samples. It would be interesting to shed some light on this question. On the other hand, no TL differences in the primary tumours within the groups of patients diagnosed with either synchronous or metachronous metastatic disease were observed. Our result is in agreement with Le Balc’h et al. who also did not find any association between TL in tumours of patients with CRC and the presence or absence of distant metastasis at the time of diagnosis^17^. However, in a study by Ye et al. patients diagnosed with advanced CRC with distant metastases had shorter telomeres in primary tumours compared to those of non-metastatic patients^22^.

Herein, we also documented a correlation between TL and the size of the metastatic lesion. To our best knowledge, no studies have described similar results in patients diagnosed with CRC. Studies focusing on different tumour types, including 348 and 140 breast cancer patients, did not find a similar relationship in the tumour tissue of the patients^31,32^. Another study showed a correlation between reduced TL in either tumour tissue or cancer-associated fibroblasts and increased tumour size in patients diagnosed with hepatocellular carcinoma^33^. The inconsistency in the results needs further investigation since the findings might be specific for particular cancer types.

In our study, the patients diagnosed with rectal cancer following the neoadjuvant therapy had significantly shortened telomeres in either primary tumour tissue or non-cancerous mucosa than in the same tissues from the same subjects before the therapy treatment. As documented previously, TL shortening in the non-cancerous mucosa could be predictive of treatment-related toxicity^34,35^. During neoadjuvant therapy, malignant cells, inclusive non-cancerous, are exposed to radiation therapy (RT) and chemotherapy. Telomeres are particularly susceptible to RT-induced damage owing to their sensitivity to reactive oxygen species arising during RT^34,36^, which may lead to TL shortening^37^. Additionally, some cytotoxic drugs can cause TL shortening by direct DNA damage or secondary-induced oxidative stress^38^. The current body of research on telomerase activity in relation to radiation has yielded conflicting results. While it has been suggested that radiation exposure up-regulates telomerase activity specifically in cancer cells^39^, another study found decrease in telomerase activity in cultured human lens epithelium cells following different doses of ionizing radiation^40^. Given this inconsistency, it is clear that further investigation is needed to fully understand the complex interplay between telomerase and radiation. Assuming that telomeres are considered hotspots of high sensitivity to RT, both TL, and telomerase activity may be promising biomarkers of radiosensitivity^41^. The ratio between TL in the tumour and adjacent non-cancerous mucosa might provide valuable information reflecting potential treatment benefits, but due to the limited sample size, this study was not powered to evaluate the potential predictive effect of TL on response to neoadjuvant therapy.

In the present study, we could not observe any differences in TL within the primary tumour between patients with mutant and wild-type *KRAS* status, respectively. Like dysfunctional telomeres, *KRAS* mutations are known to drive chromosomal instability^42,43^. As previously documented, activation of the Ras/Raf/MEK/Erk pathway may lead to increased *hTERT* transcription and, therefore, to upregulated telomerase activity^44^. Our observation is in contrast to the study by Le Balc’h et al. who found shorter telomeres in individuals harbouring *KRAS* mutations (n = 42) within a group of 125 patients^17^. The authors concluded that it is currently unknown why patients with *KRAS* mutation exhibit eroded telomeres in tumour tissue. We are aware that the data about *KRAS* mutation status in our study was available for a limited number of patients and also that our studied group was much smaller than the one analysed by Le Balc’h et al*.*

A growing body of evidence suggests that TL in the tumour tissue might harbour prognostic information in patients with various solid tumors^14–16^. However, the results are often conflicting, and their interpretation might be challenging. We observed significantly increased OS in patients with a TL ratio between the tumour tissue and adjacent non-cancerous mucosa greater or equal to 0.378. It is consistent with the results of the meta-analysis by Zhang et al. where short TL in primary tumours predicted poor OS in CRC^14^, though the authors did not consider non-cancerous mucosa as a reference. However, in our previous study, a lower TL ratio between tumour and non-cancerous mucosa was associated with increased OS in CRC patients^13^, similar to the meta-analysis by Jia and Wang^1^. Several studies also found an association between short TL and high-risk tumour features (including large tumour size, a large number of lymph node metastases, vascular invasion, and tumour grade)^15,45–47^, while the opposite trend was observed in others^26,48,49^. Therefore, the association between TL and prognosis has to be interpreted with caution and other patient and tumour-related factors have to be considered. A recent study used a TL-related signature including 18 genes to stratify non-small cell lung cancer patients into low- and high-risk groups^50^. A similar telomere-related gene risk model was successfully used for predicting the outcomes of renal cell cancer patients^51^. This approach utilizes more complex information on the telomere system function and might provide more reliable prognostic information than the TL or telomerase activity alone. However, prospective validation on a large cohort of patients is warranted.

A strong feature of our study is that we were able to address TL in primary tumour tissue, bowel mucosa, liver metastases and liver parenchyma in a relatively large number of subjects. We are also aware of the limitations of our study. We reported only the average relative TL and not the percentage of critically shortened telomeres in the tissue sample, as would be possible using Telomere Shortest Length Assay (TeSLA)^52^. However, the DNA fragmentation caused by the fixation process could hinder our ability to obtain reliable measurements using TeSLA in this particular context. Alternatively, the telomeric DNA quantification by the multiplex qPCR required only small DNA input and was optimal for the study due to its use of low-concentrated FFPE-derived DNA.

## Conclusion

Telomeres play a crucial role in cancer development and progression. This study provided insights into the TL dynamics during the CRC progression from non-cancerous mucosa to primary tumours and liver metastases. We observed TL shortening in primary tumours compared to non-cancerous mucosa suggesting its important role in malignant transformation. However, no difference was observed between the TL in the primary tumour and liver metastases. Shorter TL in metachronous liver metastases suggests more aggressive behaviour of cell subpopulations with shorter TL. Supporting this hypothesis, Ceja-Rangel et al. 2016 reported that breast cancer cell lines with shorter telomeres and high telomerase activity exhibited a highly aggressive phenotype^53^. We also found a correlation between TL and metastatic lesion size. Significant variability in TL was observed in primary tumours originating from the proximal colon, distal colon, and rectum. This might be related to different embryonic origin of the right and left colon/rectum and uneven distribution of specific mutations. Neoadjuvant chemoradiotherapy leads to significant TL shortening in both the tumour tissue and adjacent normal mucosa of patients with rectal cancer reflecting high susceptibility of telomeres to RT in both the tumour and normal tissues. We also observed a significant prognostic value of the TL ratio between the tumour tissue and adjacent non-cancerous mucosa, as the higher ratio was associated with increased OS. However, as the studies reporting the association between TL and prognosis provide conflicting results, the outcomes need to be approached with caution. In this regard, TL-related gene signatures might provide more complex and reliable prognostic information and warrant further validation.

## Data Availability

The data are available in coded form at the Institute of Experimental Medicine of the Czech Academy of Sciences. The datasets used and/or analysed during the current study are available from the corresponding author on reasonable request.
